# The Pre-History of Urban Scaling

**DOI:** 10.1371/journal.pone.0087902

**Published:** 2014-02-12

**Authors:** Scott G. Ortman, Andrew H. F. Cabaniss, Jennie O. Sturm, Luís M. A. Bettencourt

**Affiliations:** 1 Department of Anthropology, University of Colorado Boulder, Boulder, Colorado, United States of America; 2 Santa Fe Institute, Santa Fe, New Mexico, United States of America; 3 Classics Department, University of North Carolina Chapel Hill, Chapel Hill, North Carolina, United States of America; 4 Department of Anthropology, University of New Mexico, Albuquerque, New Mexico, United States of America; University College London, United Kingdom

## Abstract

Cities are increasingly the fundamental socio-economic units of human societies worldwide, but we still lack a unified characterization of urbanization that captures the social processes realized by cities across time and space. This is especially important for understanding the role of cities in the history of human civilization and for determining whether studies of ancient cities are relevant for contemporary science and policy. As a step in this direction, we develop a theory of settlement scaling in archaeology, deriving the relationship between population and settled area from a consideration of the interplay between social and infrastructural networks. We then test these models on settlement data from the Pre-Hispanic Basin of Mexico to show that this ancient settlement system displays spatial scaling properties analogous to those observed in modern cities. Our data derive from over 1,500 settlements occupied over two millennia and spanning four major cultural periods characterized by different levels of agricultural productivity, political centralization and market development. We show that, in agreement with theory, total settlement area increases with population size, on average, according to a scale invariant relation with an exponent in the range 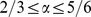. As a consequence, we are able to infer aggregate socio-economic properties of ancient societies from archaeological measures of settlement organization. Our findings, from an urban settlement system that evolved independently from its old-world counterparts, suggest that principles of settlement organization are very general and may apply to the entire range of human history.

## Introduction

Many studies over the last few decades have demonstrated that aggregate properties of contemporary urban settlements –from socio-economic outputs to land area to the extent of infrastructure– vary systematically and predictably with population size [1]–[9]. These regularities emerge from two advantages of larger settlements: the realization of greater material economies of scale, and the promotion of increased rates of social interaction, which enhance the production of general socio-economic quantities including those related to the size, organization and value of their economies. It is a question of great interest whether these functional properties were also present in ancient cities [10]–[12]. In this paper we derive several consequences of scaling theory for the study of ancient settlements and test the resulting models using archaeological data from Pre-Hispanic Central Mexico. Our results suggest the fundamental processes behind contemporary urban scaling operated in the ancient world just as they do today.

Elements of modern urban theory have often been used in interpreting archaeological evidence, especially to help understand the origins of cities and the relationship between urbanism and early states [13]–[16]. Two well-known examples are the concepts of settlement-size hierarchies and rank-size distributions [17], [18]. The first derives from central place theory [19] and is used to predict functional hierarchies of services linking the size of settled areas to levels of regional administration, distributions of public buildings, and prevalence of administrative artifacts [20], [21]. The second builds on models devised to explain Zipf's law for the relationship between the rank and size of cities in an urban system [22]–[24] and interprets deviations from the proportional rank-size rule in terms of the relative degree of system integration [25]. These concepts have been and continue to be useful but, in their present form, they have certain limitations. For example, the theoretical models underlying both approaches lead to static equilibria in the spatial and/or rank-order distributions of city sizes, and thus cannot inform on how urban systems arise, how they grow, or what political, economic or technological transformations characterize them. In addition, these ideas make no quantitative predictions about the distribution of socio-economic functions with city size, beyond the fact that they should, on average, form a hierarchy [26].

To address these shortcomings, modern theories of economic geography [27], [28] and of cities as complex systems [6] have shifted focus to the structure and function of individual settlements. A key element of these approaches is the observation of increasing returns to scale, that is, that per capita socio-economic rates, such as wages or GDP, increase with city population size in a way that is scale-invariant and, in principle, open-ended. This allows more populous cities to develop more complex social organizations with a greater range of specializations, which in turn helps explain their role in urban hierarchies [29]. However, it was only recently, as a result of comparative analyses of large datasets for many urban systems around the world, that the link between settlement area, the geometry of infrastructural networks (such as paths and roads) and socio-economic rates was firmly established [6].

This theory derives many average properties of modern cities from their population size based on a few general principles of human social organization [6] and leads to a general view of cities as *social reactors*: larger cities, on average, magnify social interaction opportunities thereby increasing the productivity and scope of material resources and human labor. This accumulation of functions with population size also provides a mechanism for the genesis of settlement-size hierarchies that characterize both ancient and modern societies [10], [11]. In this way, urban scaling theory provides a link between social, spatial and infrastructural patterns of settlement and the socio-economic roles of cities across time and space.

Here, we develop these ideas in the context of archaeology and test some of the resulting predictions using data from Pre-Hispanic Central Mexico, an urban settlement system that developed independently of its old-world counterparts. Motivated by the characteristics of the archaeological record we develop a model of settlements as social networks embedded in space. This allows us to account for the expected spatial organization of very simple small settlements and their elaboration into cities in terms of the organization of their built environments. We then test model predictions against the quantitative patterns of over 1,500 settlements spanning two millennia and four major cultural periods. We close by discussing the relevance of these results for the general understanding of cities in history and for archaeology in particular, suggesting additional ways in which scaling theory, suitably developed in archaeological contexts, can reveal aspects of the socio-economic organization of Pre-Hispanic Mexico and other ancient societies accessible to us through the archaeological record.

## Settlement Scaling Theory in Archaeology

Arguably, the most important feature of human sociality is that individuals can derive advantages from social contact, e.g. by trading goods, sharing information and developing cooperative strategies [32]. The sharing of information in cities has been emphasized since very early in economic theory [33], and has been taken up by many contemporary authors who note that cooperation is facilitated by repeated contact and by dense social networks.This means that, under general conditions [6], humans benefit from creating large interacting social networks. Such networks exist in hunter-gatherer societies, but these can be amplified and intensified by settlement, through co-location in space and in time. Thus, concepts of human social interactions embedded in space provide a simple way to formalize the expected scaling properties of human settlements.

**Figure 1:**
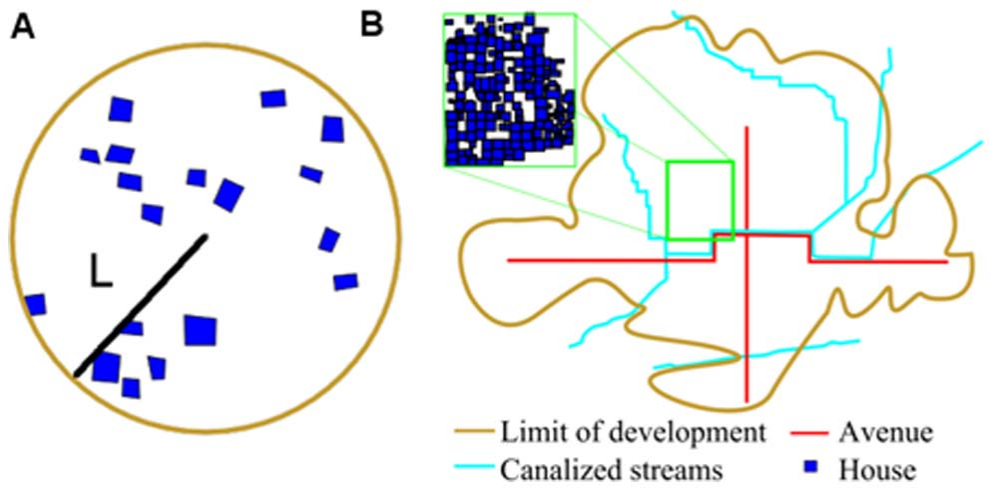
Models of settlement structure and social interactions. A. The Amorphous Settlement Model, showing a small settlement (Capilco) that can be i) easily circumscribed by a circle of radius 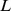 and ii) traversed primarily by linear paths [30]. B. The Networked Settlement Model, showing an infrastructure-dense city (Teotihuacan) containing large avenues (red), canalized streams (light blue), and streets connecting open spaces, apartments, and the major avenues (dark blue inset); the settlement area acquires a structured shape determined by the underlying infrastructure network [31].

Typically, small settlements appear disorganized spatially and are characterized by low population density. We call these *amorphous* settlements, which tend to provide the simplest forms of spatial organization. To understand the relationship between their occupied land area and population consider the situation where each individual maintains social interactions with others by moving within the settled area, 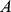. We express the distance (proportional to the settlement's diameter) covered by this movement, 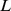 in terms of 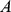 as 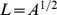, see Fig. 1A. Any individual can reach any part of the settlement by taking approximately straight paths across the unstructured and relatively sparsely built up area. Thus, the total cost of this movement, 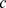 is proportional to 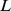, that is 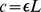, where 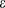 is the cost per unit time and unit length travelled, e.g. the metabolic energy expended in walking.

We can now contrast this cost to the benefits obtained through social interaction and compute the dependence of land area on population size. Assuming that the chance of social contacts is homogeneous over the settled area, the number of social contacts per person, 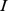, is simply proportional to population density 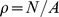, that is 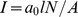, where 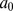 is an individual's interaction strength with others (a cross-section) and 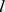 is the average length travelled per person. We translate the benefits obtained from these social interactions into units of energy per person and unit time, 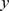, through a conversion factor 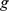, so that 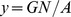, with 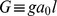. The scaling of total area with population then follows by equating benefits to costs, 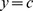, and solving for 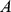 as a function of 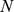. We obtain 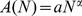, with 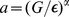, and 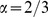 (This value for the exponent 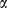 was first derived by Nordbeck [2], who observed it in modern urbanized areas in Sweden. His argument however was solely geometric: noting that population densities vary across space within a city, he suggested that population may behave as a 3-dimensional space-filling fractal. Here, we derive this same exponent from the more fundamental dynamics of human social interactions subject to movement costs).

However, the arguments that apply to small and amorphous settlements need to be modified when one considers larger and denser settlements. This is because higher settlement densities lead to increasingly structured land use, and specifically to the segregation of roads and public spaces from dwellings and other buildings. More specifically, for larger settlements it often becomes more natural to measure the built-up area instead of the circumscribing land area (see Fig. 1B). Settled area is defined by infrastructure networks, which fill up a larger fraction of the total land area in larger settlements [6]. The scaling of these infrastructure networks, which in ancient cities consisted of roads, paths and canals, obeys a different kind of organizational principle which is also observed in modern cities [6], [9]. The idea is that cities grow spatially at their margins by building decentralized infrastructure networks. This means specifically that new pieces of settled land are connected to the rest of the city by incrementally growing infrastructure in a manner that is consistent with the current overall density. Mathematically, this means that, with each net increase in population, the city grows its infrastructural area, 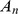, incrementally, proportionally to the average distance between people, 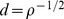, set by the current overall density 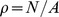, so that 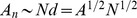. Substituting 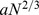 for 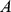, this leads to 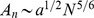 [6] (This scaling relation can also be derived from more detailed microscopic models of infrastructure, which consider energy dissipation in the network and its associated maintenance costs [6]). Thus, scaling relations characterizing how settlement area varies with population should exhibit exponents 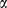 in the range 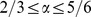, with the lower limit characteristic of unstructured settlements and the upper limit characteristic of settlements defined by infrastructure networks. If the area of infrastructure can be measured directly (as is often done in modern cities through satellite imagery [1]) then 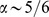 [6].

Several additional considerations are important but do not affect the fundamental range of values that 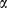 can take. First, settlements large and small may be more or less amorphous, and many forms of planning can generate a networked settlement [12]. These issues affect the pre-factors in 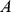 and 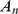, but not their exponents, as is discussed in Ref. [6]. In the same way, a variety of factors, including transportation technology and per capita production and consumption rates, are expressed via the parameters in 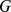 and 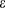 and only influence the pre-factor 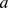, not the exponent 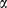 [6]. We note in addition that our model assumes that cities exist to the extent that they create and sustain large social networks and that the average rate of social interaction determines most urban socio-economic outputs. It follows that the total socio-economic output of a settlement, 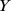, depends on its population according to a scaling relation of the form 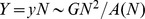, where 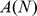 becomes 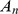 in larger settlements. Finally, it is important to emphasize that the model developed here assumes individuals explore a settlement fully, thus leading to 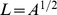. However, one can generalize this relation as 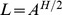, where 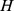 is a fractal dimension of paths representing the proportion of the transverse dimension that is actually explored. If the full transverse dimension is explored, 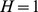 and one can proceed as above. This generalization leads to the scaling exponent 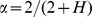 for amorphous settlements and 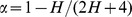 for networked settlements. If 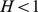, as might happen in cities that are very segregated spatially or socially, individuals explore increasingly smaller fractions of the city overall, and the area-population relationship becomes increasingly linear (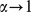), to the point that large settlements provide no additional benefit [6]. We discuss this generalized model with respect to the degree of social and spatial integration in ancient cities below.

### 2.1 Expectations for the Archaeological Record

The theoretical framework developed above applies to settlements for which it is reasonable to model the settled area as the *container* within which a resident population interacts on a regular basis. It proposes that such settlements tend to grow in ways that balance the costs of moving within the settlement with the benefits of the resulting social interactions. Thus, if the costs of movement and the average benefits of social interaction are constant in a given context, settlements whose spatial arrangements are designed to balance these costs and benefits should exhibit a specific and consistent overall relationship between the resident population and settled area. Specifically, the settled area should increase more slowly than the resident population such that, on average, a doubling of population only leads to a 2/3 increase in settled area for small amorphous settlements and a 5/6 increase for larger, networked settlements. Thus, the first predictions of our model are that the exponent relating population to settled area for “interaction container” settlements in a given context should fall in the range between 2/3 and 5/6, with this exponent being closer to 2/3 among small, amorphous settlements and closer to 5/6 among larger, networked settlements. In other words, our theory predicts that, as human settlements grow, they get denser in a context-specific but mathematically-predictable way.

Our framework also predicts that the area taken up by an individual in the smallest such settlements derives primarily from travel costs and the average benefit of social interactions. In ancient societies, these parameters were defined by the energy expended in walking and the energetic benefit conveyed by socio-economic exchanges. Technologies that reduce transportation costs or increase the effectiveness of interaction should exert a significant influence on this baseline area, but factors that influence the way in which a society captures energy (e.g., agricultural technology and political organization) should not. These factors do define an upper limit for settlement sizes in a given context (see, e.g. [36]), but they should not influence the baseline area taken up by an individual in the smallest “interaction container” settlements. Thus, a second prediction of our models is that the pre-factor of the scaling relation between population and settled area should be responsive to changes in within-settlement transport technology and to influences on the flow of goods and services between people, but not to changes in agricultural productivity or political organization per se.

## Materials and Methods

### 3.1 Data Sources and Population Estimates

**Figure 2:**
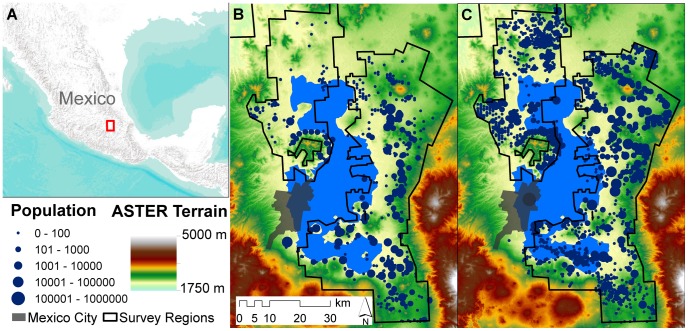
Maps of the Basin of Mexico. A: Location within Mexico [34]. B: Settlements dating to the Formative period (circle size is proportional to population; colors range from yellow through red to white denoting increases in elevation; gray area shows the extent of Mexico City in 1964) [35]. C: Settlements dating to the Aztec period. During the latter period settlement expanded into the shallows of the lake. Today, settlement covers the entire basin and the lake has been drained.

We test the expectations above using settlement data from archaeological surface surveys conducted in the Basin of Mexico (BOM) between 1960 and 1975 by The University of Michigan and Pennsylvania State University (Figure 2A). These surveys produced a remarkably-complete documentation of the Pre-Hispanic settlement system of this region prior to the destruction of many sites by the expansion of modern Mexico City. Survey data from this work are available at http://www.lsa.umich.edu/umma/collections/archaeologycollections/latinamericanarchaeology, and on a CD included with [43]. Working from these digital sources and primary survey reports [37]–[47] we compiled the following data for each of the approximately 4,000 sites recorded by the surveys: 1) the settled area; 2) the median density of surface potsherds within the settled area; 3) the count and total surface area of domestic architectural mounds; 4) the settlement type into which each site was classified; 5) the population estimate; and 6) the time period. We also added data for a few important sites outside the surveyed area (Cuicuilco, Tenochtitlán-Tlatelolco, and Tenayuca) and for Teotihuacan based on information in the literature (For Teotihuacan, the total residential mound area was estimated by multiplying the number of apartment compounds present at the site (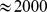) by their mean area (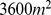); and the house-counting population (see below) was estimated by multiplying the number of apartment compounds by the estimated number of inhabitants of an average-sized compound (60 persons), following [21], [48]–[50]). The resulting dataset is available at http://www.tdar.org/.

BOM surveyors estimated the settled area by outlining the distribution of surface remains dating to a particular period on low-altitude aerial photos, and they categorized surface potsherd densities within these areas according to the scheme described below. When surface architectural remains were preserved, surveyors also interpreted the original function of each mound (civic-ceremonial, domestic residence, or salt-making) and estimated the area and height of each.

Surveyors estimated population for each period of occupation at each site using one of two methods. When architectural remains were well-preserved, surveyors worked from the count or total surface area of residential mounds in combination with excavation results. For sites dating to the Classic Period (100 B.C.E.–750 C.E.), excavations indicated that each residential mound was home to several domestic groups, so the estimation method for these sites was to multiply the mound area by.55 (the proportion of the pre-excavation surface area that proved to consist of residential space) and then divide the result by 30 (the square meters of residential space utilized by each person in sites of this period) [45]. For sites dating to other periods, excavations indicated that each residential mound was home to a single domestic group averaging 5–10 persons, so the method adopted for these sites was to multiply the total number of residential mounds by 5–10 [41], [46], [54].

In the majority of cases, architectural remains were not preserved sufficiently to apply house-counting methods in their population estimates. For these sites, surveyors devised an alternative method that involved: 1) defining the extent of the surface artifact scatter for each period of occupation; 2) assigning each scatter to one of a series of artifact density classes; and 3) multiplying the extent of the scatter for each period by a population density derived from associations of surface potsherd densities with population densities of various settlement types in 16th and 20th century records from the area. The density classes and conversions used in this method are as follows (see [21], [37]):

- Very Light – A wide scattering of surface debris so that only one or two sherds may be present every few meters; associated with compact rancherias of 2–5 persons/ha.
- Light – A continuous distribution of sherds every 20–30 cm, but with no significant build-up in sherd density beyond that point; associated with scattered villages of 5–10 persons/ha.
- Light-Moderate – Although most of the area contains light surface remains, delimited areas of substantial buildup containing as many as 100–200 sherds per square meter consistently appear; associated with compact low-density villages of 10–25 persons/ha.
- Moderate – A continuous layer of sherds, so that any randomly placed 1-meter square might yield 100 to 200 pieces of pottery; associated with compact high-density villages of 25–50 persons/ha.
- Moderate-Heavy – Over most of the area occupation occurs in moderate densities, however, in a few localized areas a 1-m square might contain 200–400 pieces of pottery; upper range of compact high-density villages of 50–100 persons/ha.
- Heavy – Densities of 200–400 sherds per 1-m square are continuous such that sherds are literally one atop another, so that a randomly placed 1-m square might produce as many as 400–800 pieces of pottery; associated with the upper range of compact high-density villages of 50–100 persons/ha.

In cases where different densities occurred in sub-areas of a single period of occupation at a single site, surveyors generally estimated population separately for the sub-area associated with each density class and then summed the results for that period [21], [42].

The digital compilations and primary sources were not always explicit regarding the estimation method used for specific sites, so we compared the population estimate of each site with its settled area, surface potsherd density class and recorded architectural remains to isolate those sites for which house-counting was used. This analysis determined that house-counting was used to estimate the populations of about 5 percent of the recorded sites. Most of these are farming hamlets containing 1–2 mounds, but several larger settlements, including the Classic Period metropolis of Teotihuacan, are also included in this list. We also identified a small number of sites at which architectural remains were abundant but population was estimated using the area-density method, or architecture was preserved in only a portion of a site and the house-counting population of this portion was extrapolated to the remainder of the site area. The sites identified through this process play an important role in our analyses below. Surveyors typically presented population estimates as ranges and as a most likely value; we took the most likely value in the final data compilations as the best estimate for the average resident population of each site during a given period. Given the nature of the archaeological record and the methods used one would expect significant errors in the population estimates for individual sites. However, so long as these errors are relatively unstructured and are not settlement size dependent these data should be adequate for estimating underlying average scaling relations and determining whether these are within the range of theoretical expectations given above.

### 3.2 Settlement Selection and Grouping

Basin of Mexico (BOM) surveyors classified each site into a series of settlement types based on the spatial extent, density, and character of the surface remains. Because our theory suggests scaling relations arise from the interactions of residents within settlements, we excluded site types that do not conform to the “interaction container” model, namely: 1) sites lacking permanent residential populations, such as isolated ceremonial centers, quarries and salt mounds; and 2) dispersed sites consisting of isolated residences interspersed with farmland [21]. We also excluded sites with settled areas less than 1 ha from our analyses due to limits in the precision of the recorded data which hinder scaling analysis of these smallest sites.

We grouped the remaining ca. 1,500 settlements in two ways. First, we created four 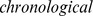 groups by assigning each site to one of four time periods dating from initial colonization of the Basin up to the Spanish Conquest, following the chronology in the most recent publications of these data [40], [42]. The Formative period (1150 B.C.E.–150 B.C.E.; Figure 2B) saw the beginnings of detectable settlements and the rise of local polities; the Classic period (150 B.C.E.–650 C.E.), the political and economic dominance of Teotihuacan (ca. 100,000 people); the Toltec period (650–1200 C.E.), the formation of a number of small competitive polities; and the Aztec period (1200–1519 C.E.; Figure 2C), the unification of these into an empire centered on Tenochtitlán-Tlatelolco (ca. 200,000 people). It is important to note that the sites included in each group were not strictly contemporaneous and in some cases derive from multiple socio-political units, but these issues are not relevant here because the theory we test involves patterns in the use of space within settlements as opposed to networks of relationships between them. Second, we created two 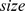 groups by distinguishing settlements with 5,000 or more people from smaller settlements. The break point of 5,000 corresponds to the typical population size of Aztec city-state capitals defined in previous work [49], [55] and is made so as to distinguish smaller amorphous settlements from larger networked settlements where infrastructure should be more prevalent.

### 3.3 Scaling Parameter Estimation

We calculated scaling parameters, 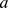 and 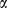, for each chronological group and size group using two standard estimation methods. First, we applied ordinary least-squares regression (OLS) to the log-transformed data. Second, we produced maximum likelihood estimates (MLE) by iteratively maximizing the log-likelihood of the scaling parameters, given the data, until they converged upon a (local) maximum. MLE does not overly weight outliers as least squares regression may and has been considered the most robust method for defining power law behavior [51]. In this case, the area 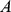 is assumed to be log-normally distributed with mean 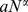 (where 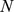 is population, 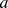 is a coefficient and 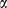 is a scaling exponent) and a standard deviation defined from the distribution of residuals from OLS regression of the log-transformed data for each group. We also calculated confidence intervals for both the OLS and MLE estimates using a bootstrap procedure. Bootstrapping re-samples from the sample distribution with replacement to approximate draws from the original population [52]. By repeating this process many times (in our case 1000) and fitting parameters each time, we produce probability density distributions for the parameters in question. The confidence interval is simply the middle 95% of this distribution. The resulting estimates and confidence intervals are presented in Tables 1 and 2.

**Table 1:** Scaling relations for settled area versus population in the BOM.

Group:	Formative	Classic	Toltec	Aztec
N sites	230	272	484	546
MLE 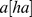	.200	.274	.196	.180
95% C.I.	.174–.277	.206–.400	.167–.256	.154–.230
MLE 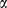	.708	.573	.715	.731
95% C.I.	.654–.736	.507–.654	.674–.763	.702–.777
OLS 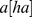	.235	.294	.215	.195
95% C.I.	.198–.277	.214–.407	.184–.253	.175–.222
OLS 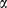	.700	.627	.708	.750
95% C.I.	.654–.740	.544–.705	.655–.752	.714–.785
Magnitude	33,850	95,597	22,502	212,500
Centrality	.295	.620	.229	.350
Productivity	700	1,400	1,400	3,000

Estimated pre-factors 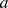 and exponents 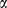 for four Pre-Hispanic periods. Parameter estimates and 95% confidence intervals (CI) obtained via maximum likelihood estimation (MLE) and ordinary least squares minimization (OLS). *Magnitude* is the estimated population size of the largest settlement, *Centrality* its fraction of the total population, and *Productivity* the yield (kg maize/ha) of the most productive agricultural strategy [53].

**Table 2:** Scaling relations for settled area versus population among amorphous vs. networked (population 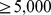) settlements in the BOM, and for the 1960 census in the same region.

Group:	Amorphous	Networked	1960
N sites	1510	22	181
MLE 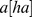	.265	.294	.365
95% C.I.	.220–.285	.001–5.45	.206–.925
MLE 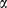	.652	.724	.601
95% C.I.	.626–.674	.434–1.135	.493–.706
OLS 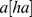	.237	.109	.445
95% C.I.	.217–.259	.009–1.303	.250–.945
OLS 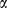	.671	.853	.641
95% C.I.	.651–.691	.598–1.109	.552–.729
OLS 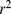	.741	.709	.532

Estimated pre-factors 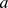, exponents 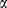 and 95% confidence intervals (CI) obtained via maximum likelihood estimation (MLE) and ordinary least squares minimization (OLS).

### 3.4 Data Validation

It is important to emphasize the potential problems raised by the area-density method of estimating population as it leads to estimates that derive in part from settled areas, thus ensuring that settled area and population will exhibit a systematic relationship. The question, however, is not whether a systematic relationship between settled area and population exists, as this is to be expected regardless of the estimation method used. Instead, the critical question is whether the specific functional form and parameter values predicted by our theory are a necessary outcome of the area-density method used to estimate population for most sites in the BOM surveys. We addressed this issue in a number of ways.

First, we performed Monte Carlo simulations to determine the scaling relations that would be expected if artifact densities, and their corresponding population densities, varied independently of site area. This allows us to test whether the scaling relations we observe in the data could have emerged as a result of other factors that may affect surface artifact visibility. Second, we compared the Pre-Hispanic data with 1960 census data from the same region, as reported in various BOM survey volumes [37], [38], [40], [41]. This allows us to assess whether the specific exponent and prefactor values we estimate for the Pre-Hispanic periods are reasonable in light of those reflected in the most recent period of primarily non-industrial settlement in the area. Third, we performed a number of analyses of the data from sites with well-preserved architectural remains. These allow us to test whether the scaling relations we observe in the larger dataset are also apparent among the subset of sites where population was estimated using house-counting methods. Results of these analyses are discussed below.

## Results

Table 1 presents our estimates of the parameters of scaling relations for settlements dating to each of our four chronological periods. In almost all cases we observe clear sub-linear scaling (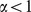) of settlement area with population within the expected range derived above. The lone exception is the Classic period, where the confidence interval of the MLE estimate of 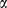 does not encompass 2/3, meaning that population density may have increased more rapidly with population size during the Classic period than expected, even for amorphous settlements. This may be due to the fact that most settlements of this period were in the Teotihuacan Valley, that this was the first area surveyed, and that the BOM survey methods were first worked out in this area and may not have been applied as consistently [43]. Regardless, the results in Table 1 still demonstrate the overall trend of increasing population density with settlement population (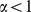) for settlements ranging from small farming hamlets of 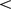
10 people to the Aztec Imperial Capital of Tenochtitlán-Tlatelolco (with a population 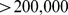). It is also important to note that the Aztec period presents exponents closest to 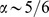, consistent with expectations for settlements exhibiting shapes set by infrastructure related to interaction such as plazas, marketplaces and roads, which were most widespread during this period [49], [57]. Overall, these findings are consistent with the first expectation of our theory and support its assertion that human settlements of all scales function in essentially the same way by concentrating social interactions in space. In addition, we observe that scaling pre-factors and exponents are not correlated with measures of political centralization or agricultural productivity, as shown in Table 1. During the Pre-Hispanic era, political centralization waxed and waned and agricultural productivity quadrupled, but relationships between population and settled area remained remarkably consistent. Given that movement within settlements was exclusively pedestrian throughout the Pre-Hispanic Period, this finding is consistent with our second expectation and supports a key assertion of our theory; namely that relative economies and returns to scale emerge primarily from the balancing of transport costs and interaction benefits within settlements as opposed to specific agricultural technologies or forms of political organization. This appears to be as true for ancient settlements as it is for modern cities.

Although sublinear scaling of settled area with population was relatively consistent through time, the scaling parameters estimated for each period still do vary somewhat. In light of our prediction that the exponent of the average scaling relation should shift from 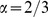 to 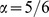 as settlements grow, one possible source of this variation is the size distribution of settlements assigned to each chronological group. Table 2, which estimates scaling relations for our two size groups, supports this possibility. These analyses show that the average scaling relation for all settlements with fewer than 5,000 residents is well-described by a power law with exponent 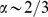, and that the average relation for larger settlements is equally well-described by a power law with exponent 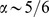. These results provide a partial explanation for the variation in scaling parameters in Table 1 and support our expectation that the rate at which settled area increases with population should change from 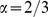 to 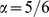 as the populations of settlements grow and the settled area becomes arranged around infrastructure networks.

Our validation analyses further demonstrate that the scaling relations in Tables 1 and 2 are not a by-product of the area-density method used to estimate population for most sites in the BOM surveys. First, Monte Carlo simulations demonstrate that the scaling relations observed in the data could not derive from an archaeological record where surface artifact density varied independently of settled area. We randomly assigned one of the population density classes used in the surveys to 1000 site areas, chosen randomly from the overall dataset. Then, we used the surveyors' conversions to calculate populations for those areas and estimated the scaling exponent for this “dummy” dataset using OLS. We then performed this procedure 1000 times and used the results to construct a 95% confidence interval for the exponent we would expect if surface artifact density varied independently of site area. The resulting distribution has a mean of 1.00, and a 95% confidence interval ranging from.95 to 1.05. Thus, if surface artifact densities varied independently of site area we would expect the exponents in Tables 1 and 2 to approach a linear relation, 1.00. As the exponents estimated from the actual data are all outside this range, we conclude the observed scaling relations could not have resulted from an archaeological record where artifact density varied independently of settled area, for whatever reason.

**Figure 3:**
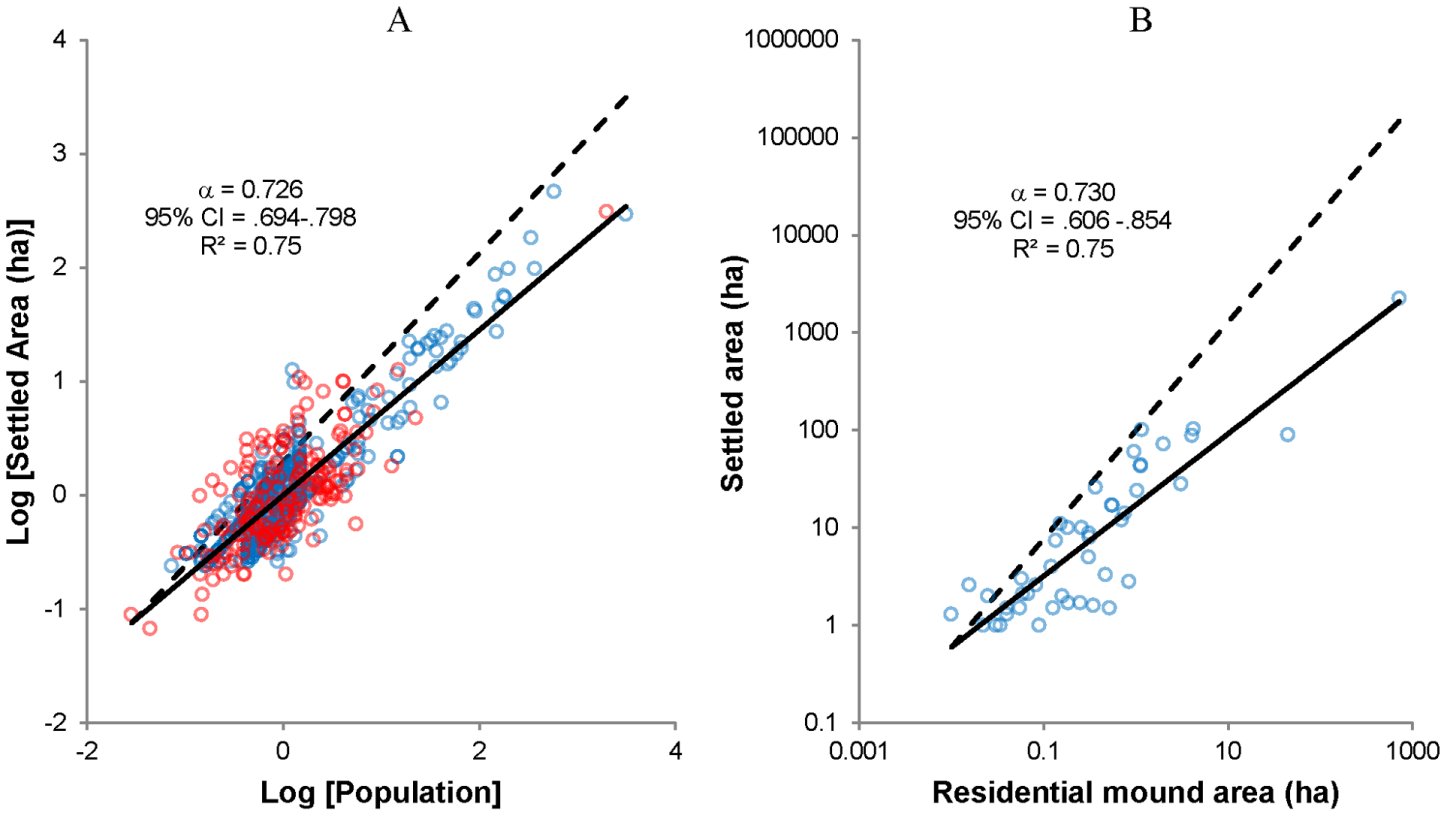
Scaling of settled area with population. A. Population vs. Settled Area for Aztec (blue, archaeological data) and 1960 (red, census data) settlements; for display, the data series have been centered by subtracting the average scaling relation in logarithmic variables, 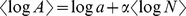, from both datasets, so that the Aztec and 1960 census data share the same average coordinate on both axes; for power-law fits for the individual data series, see Tables 1 and 2; B. Residential mound area vs. settled area for sites with well-preserved architectural remains; also see Table 3. In both charts the annotations present power-law fits from OLS regression of the log-transformed data, solid lines represent power-law fits of the displayed data and dashed lines represent proportionate (linear) scaling.

Second, comparisons with recent census data from the same region illustrate that the specific parameter values we estimate for the Pre-Hispanic periods are reasonable. The right-most column of Table 2 contains estimates of scaling exponents and prefactors for settlements recorded in the 1960 census of the BOM. Since, according to our theory, the scaling prefactor 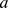 is responsive to transportation technology (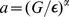, where 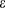 is the cost per unit time and unit length travelled), one would expect this parameter to have been somewhat larger in 1960 than it was during the Pre-Hispanic periods. Table 2 shows that this is in fact the case. In contrast, our theory suggests that scaling exponents should be similar across contexts, and this is also borne out in Table 1 and 2 and in Figure 3A, which illustrates that Aztec-period settlements and mid-20th century settlements both exhibit sub-linear scaling with comparable exponents within the predicted range. It is also important to note that, although the population of Mexico City (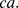 2.6M) is several orders of magnitude greater than the second-ranked settlement (Xochimilco, 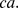 30K) in the 1960 census data, removal of Mexico City has a limited effect on the overall results (for example, OLS 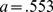 and 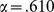 when Mexico City is removed, compare with Table 2). One could argue that the BOM survey simply mapped the population-area relationship of mid-20th century settlements onto the archaeological remains, but the Aztec period data derive primarily from correlations of surface potsherd densities with population densities, as opposed to a direct mapping of population densities onto site areas [21]. In addition, previous comparisons of 16th century colonial documents and mid-20th century census data have demonstrated similar population densities for settlements of various types during both periods [21], [54], and a direct mapping of recent population-area relationships onto the archaeological remains would not have led to the observed differences in scaling prefactors that our theory accounts for readily. Thus, the best explanation for the similarities and differences between the Pre-Hispanic periods and the 1960 census is that both systems reflect the same general properties of human settlement organization.

Third, Table 3 presents regression analyses of various subsets of the survey data which demonstrate that the area-density method used to estimate population for the majority of sites accurately captures average scaling relations in the Pre-Hispanic BOM. Models 1 and 2 assess the relationship between area-density and house-counting estimates for those cases where one method was used in the source data but the other can also be applied. Both models show that the two methods produce estimates that are strongly correlated and proportional (i.e. 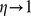 and 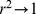), and in many cases nearly identical. Model 3 assesses the relationship between population (estimated using either method in the source data) and total residential mound area for those sites where architectural remains are well-preserved. This model shows that mound area is also strongly-correlated with and proportional to population, even among sites where population was estimated using the area-density method. Finally, Model 4 assesses the relationship between residential mound area and total settled area in sites where surface architecture is well-preserved and house-counting was used to estimate population. This model, which is also plotted in Figure 3B, exhibits the same mathematical relationship observed in the analysis of population and settled area, even though the area-density method is not involved in this case (compare with Tables 1 and 2 and Figure 3A). Given that residential space, and thus residential mound area, is also proportional to population, Model 4 demonstrates that larger settlements in the Pre-Hispanic BOM had higher population densities, and that these densities varied in accordance with the functional form and parameter values predicted by our theory and observed in the larger dataset. These analyses are critical because they demonstrate that the consistent scaling relations we observe are *independent* of the method used to estimate population.

**Table 3:** Comparisons of population estimation methods for various subsets of the BOM survey data.

Model	Independent variable	Dependent variable	Selection criteria	N	Exp.	95% C.I.	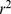
1	Population	House-counting population	Population from area-density, architecture preserved	44	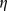 = .981	.873–1.089	.887
2	Population	Area-density population	Population from house-counting	43	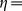 .977	.885–1.069	.917
3	Population	Mound Area	Architecture preserved	51	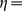 1.047	.923–1.171	.782
4	Mound Area	Settled Area	Population from house-counting, architecture preserved	46	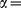 0.730	.606–.854	.761

All variables are taken in 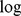 form. Included sites date primarily from the Classic and Aztec periods but all four periods are represented. Scaling exponents (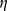, 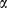) for the relationship between measures of population and the area-population relation, respectively. Standard errors and model fits (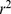) are calculated using ordinary least-squares (OLS) regression of the log-transformed data.

These analyses demonstrate that the various methods used to estimate population in the BOM surveys produced consistent results. This in turn suggests they are reasonably-accurate in an absolute sense. However, it is important to note that even if these estimates are only accurate in a relative sense they would not affect the accuracy of scaling exponent calculations; they would only affect the accuracy of prefactor calculations. We also note that, although Teotihuacan is an outlier with respect to the populations and settled areas of other Classic period settlements, removal of this site has little effect on the resulting analyses. In other words, the data from Teotihuacan are consistent with patterns apparent in smaller sites, and do not define these overall patterns.

## Discussion and Conclusions

Sublinear scaling of infrastructure and superlinear scaling of output appear to be general characteristics of contemporary urban systems, but models recently-developed to explain these patterns [6] do not invoke the specific technologies, political organizations or economic institutions characteristic of the modern world. Thus, a surprising expectation of these models is that the scaling relations observed in contemporary cities should also be apparent in ancient settlement systems. We have tested this notion here and found that Pre-Hispanic settlements in the Basin of Mexico do in fact exhibit scaling relations with population size analogous to those observed in modern cities. These relations span the urban-nonurban divide, over five orders of magnitude in settlement population, and across four cultural periods spanning more than two millennia. These results, from a settlement system that evolved independently from its old-world counterparts and which experienced significant technological, political and economic change over time, suggest that quantitative patterns of urban scaling are quite general and potentially apply to the entire range of human settlements, past and present.

Indeed, our results suggest the fundamental processes behind the emergence of scaling in modern cities have structured human settlement organization throughout human history, and that contemporary urban systems are best-conceived as lying on a continuum with the smaller-scale settlement systems known from historical and archaeological research. Our results also add support to the specific models developed in [6], and adapted to an archaeological context here, concerning the origins of scaling in cities. Specifically, they are consistent with the theoretical assertions that all human settlements function in essentially the same way by manifesting strongly-interacting social networks in space, and that relative economies and returns to scale (*elasticities* in the language of economics) emerge from interactions among individuals within settlements as opposed to specific technological, political or economic factors. Finally, our results demonstrate that archaeological data provide a useful, if generally untapped, resource for investigating scaling phenomena in human societies and that such data may shed light on the emergence and dynamics of modern, as well as ancient, urban systems [57].

The general theoretical framework developed here has significant potential for a range of applications in archaeology. For example, our findings suggest a new method for estimating the populations of archaeological sites based on their settled areas. A traditional method used in many parts of the world [20], [58] is to multiply site areas by a *constant* population density that is invariant across settlement sizes. Our results suggest this method will systematically underestimate the resident populations of larger settlements, resulting in smaller regional populations and potentially lower expectations for the level of social, political and economic organization these systems might have achieved. The area-population scaling relation can be rearranged to yield an equation that estimates the expected population of settlements, given settled areas, as 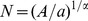, where 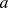 is measurable as the average area per person in the smallest settlements in the system (as 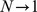), and the equation can be evaluated for 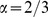 and 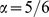 to generate a type of confidence interval. Because 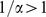 this method will lead to site populations that increase faster than their settled areas. Thus, the approach developed here leads to a refined method for reconstructing settlement patterns, rank-size distributions, site-size hierarchies, and demographic trajectories in ancient societies.

Our framework also provides a means of measuring changes in the benefits of social interaction vs. transportation costs in the past. Power-law scaling relations are characterized by two parameters, the pre-factor and the exponent, 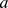 and 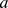 in the case of land area. The pre-factor 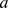 has both an immediate empirical meaning as the land area settled by an individual in the smallest settlement and the value it acquires as a consequence of the global requirement to maintain settlements connected socially 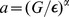. As such, 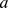 also provides a measure of the ratio of the strength of social interactions that occur in a settlement, 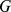, to the cost of movement, 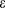. This suggests settlements may exist on different baseline scales 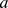 that make their fundamental density (but not its relative variation with settlement size) very different as a result of transportation technologies that affect 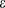, and social and political institutions that enable 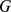 to be larger or smaller. In the BOM we find values of 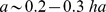 (Table 1) that are relatively consistent across cultural periods. This suggests that the ratio of social interaction benefits to transportation costs did not change appreciably over time. However, in the census of 1960, 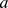 is almost twice as large as it had been in the pre-Hispanic periods, suggesting that modes of social interaction and certainly transportation technologies had changed in the direction of creating greater social incentive for interaction and/or less costly movement.

With a few additional assumptions, one can also use settlement scaling relations, together with our model, to estimate the net value of social interactions (in units of energy per unit time) in ancient societies like the BOM. For example, given that Pre-Hispanic people travelled through settlements on foot, and that on level terrain a healthy adult carrying a 30 kg load could traverse 4.5 km/hr while expending an additional 187 calories relative to sitting still [59], we obtain an estimate for 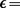
41.55 cal/(km hr). This, in combination with an estimate of 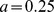 ha from our analysis of amorphous settlements and 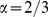 from our models, allows us to estimate 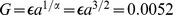 cal/(hr km^2^). This in turn allows us to estimate 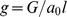 = 5.2 cal/h (assuming 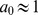 m and 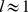 km), which translates into an average caloric benefit of 62 calories per 12 hour day. Finally, given that the total interactions, and thus the energetic benefit, experienced by an individual scales with population as 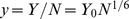, the average benefit derived from social interaction for an individual living in Teotihuacan (ca. 100,000 people) would have been approximately 425 calories per 12 hour day. This figure is equivalent to about 20% of an adult's daily caloric need, and implies that as much as 20% of the total metabolic energy expended at Teotihuacan could have been devoted to activities independent of food production. This compares favorably with estimates of the proportion of total labor input (70–90%) devoted to primary food production in other early civilizations [56].

The other parameter in the scaling relation, the exponent 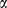, offers additional insights into the social and spatial structure of settlements. As discussed above, in our framework 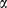 depends both on how spatial organization is achieved and on the mobility patterns of individuals within settlements. It is natural, and widely observed, that very small settlements are more spatially amorphous, meaning that they need not have a clear network of paths, roads and other public spaces to channel human movement and interaction. In contrast, larger cities like Teotihuacan and Tenochtitlán provide some of the most famous examples of the structuring of urban space in early civilizations, and their form is largely set by the network of roads and paths. We note that, assuming full mixing of resident populations, the theoretical range of the scaling exponent is 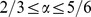. However, exponents larger than 2/3 can also derive from less-than full mixing of population, which one can account for by substituting 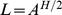 for the typical path length, and by taking 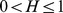 as the proportion of the transverse dimension of a settlement explored by the average individual. In this way, scaling exponents measured from archaeological evidence coupled with characterizations of settlement spatial organization can lead to interesting hypotheses about the internal structure of ancient cities. For inferred values of 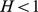, for example, one would expect settlements to be more weakly mixing, and for more isolated (and possibly segregated) neighborhoods to emerge. Thus, our framework provides a new means of investigating the internal structure of ancient cities, a topic of growing interest in archaeology [60]. These examples illustrate that our framework provides a number of opportunities for contextual interpretation of the parameters of scaling relations, a topic not pursued in [6]. Such analyses may prove useful for comparative studies and potentially provide additional tests of these models.

Finally, the framework developed here leads to exciting and testable predictions regarding a variety of socio-economic processes in ancient societies. For example, in our framework the total socio-economic outputs of settlements scales with population according to the scaling relation 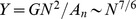 for the case where 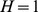. A variety of socio-economic quantities of contemporary cities, including GDP, patents, and violent crime have previously been shown to scale with population in this manner [6], [61]. In addition, our framework predicts a systematic relationship between settlement size and the division of labor, which in modern cities is reflected in the total number and degree of specialization of professions [29]. Future research could focus on developing archaeological proxy measures for socio-economic outputs and economic specialization, such as public monument construction or craft production, as a means of further testing these ideas and assessing the scope of their application. That additional socio-economic properties of ancient cities may become accessible through the interpretation of archaeological data in light of developments in urban scaling theory provides an exciting prospect not only for understanding ancient human societies but also for an integrated conceptualization of the mechanisms of socio-economic development in the past and present.

## References

[1] Angel S, Parent J, Civco DL, Blei A, Potere D (2011) The dimensions of global urban expansion: Estimates and projections for all countries, 2000–2050. Progress in Planning 75: 53107.

[2] Nordbeck S (1971) Urban Allometric Growth. Geografiska Annaler 53: 54–67.

[3] Batty M (2008) The Size, Scale, and Shape of Cities. Science 319: 769–771. 18258906 10.1126/science.1151419

[4] Sveikauskas L (1975) The Productivity of Cities. Quarterly Journal of Economics 89: 393–413.

[5] Glaeser EL, Sacerdote B (1999) Why is There More Crime in Cities? Journal of Political Economy 107: S225–S258.

[6] Bettencourt LMA (2013) The Origins of Scaling in Cities. Science 340: 1438–1441. 23788793 10.1126/science.1235823

[7] Bettencourt LMA, Lobo J, Helbing D, Kuhnert C, West GB (2007) Growth, Innovation, Scaling, and the Pace of Life of Cities. Proceedings of the National Academy of Science of the USA 104: 7301–7306. 10.1073/pnas.0610172104 PMC1852329 17438298

[8] Bettencourt LMA, Lobo J, Strumsky D (2007) Invention in the City: Increasing Returns to Patenting as a Scaling Function of Metropolitan Size. Research Policy 36: 107–120.

[9] Samaniego H, Moses ME (2009) Cities as organisms: Allometric scaling of urban road networks. Journal of Transportation and Land Use 1: 2139.

[10] Childe VG (1950) The Urban Revolution. The Town Planning Review 21 (1) 3–17.

[11] Mumford L (1961) The City in History: Its Origins, Its Transformations, and Its Prospects. New York: Harcourt, Brace & World.

[12] Smith ME (2007) Form and Meaning in the Earliest Cities: A New Approach to Ancient Urban Planning. Journal of Planning History 6 (1) 3–47.

[13] Adams RM (1966) The Evolution of Urban Society. Chicago: Aldine.

[14] Feinman GM, Marcus J, editors (1998) Archaic States. Santa Fe: School of American Research Press.

[15] Marcus J, Sabloff JA, editors (2008) The Ancient City: New Perspectives on Urbanism in the Old and New World. Santa Fe: School for Advanced Research Press.

[16] Yoffee N (2005) Myths of the Archaic State: Evolution of the Earliest Cities, States and Civilizations. Cambridge: Cambridge University Press.

[17] Johnson GA (1977) Aspects of Regional Analysis in Archaeology. Annual Review of Anthropology 6: 479–508.

[18] Wright HT, Johnson GA (1975) Population, Exchange and Early State Formation in Southwestern Iran. American Anthropologist 77 (2) 267–289.

[19] Christaller W (1933) Die Zentralen Orte in Süddeutschland. Jena, Germany: Gustav Fischer Verlag.

[20] Johnson GA (1987) The Changing Organization of Uruk Administration on the Susiana Plain. The Archaeology of Western Iran: Settlement and Society from Prehistory to the Islamic Conquest. Hole F, editor. Washington, DC: Smithsonian Institution Press. pp 107–140.

[21] Sanders WT, Parsons J, Santley RS (1979) The Basin of Mexico: Ecological Processes in the Evolution of a Civilization. New York: Academic Press.

[22] Zipf GK (1949) Human Behavior and the Principle of Least Effort. Cambridge, MA: Addison-Wesley.

[23] Simon HA (1955) On a Class of Skew Distribution Functions. Biometrika 42 (3/4) 425–440.

[24] Cristelli M, Batty M, Pietronero L (2012) There is More than a Power Law in Zipf. Scientific Reports 2 (812) 1–7. 10.1038/srep00812 PMC3492871 23139862

[25] Johnson GA (1980) Rank-Size Convexity and System Integration: A View from Archaeology. Economic Geography 56 (3) 234–247.

[26] Mori T, Nishikimi K, Smith TE (2008) The Number-Average Size Rule: A New Empirical Relationship Between Industrial Location and City Size. Journal of Regional Science 48: 165–211.

[27] Fujita M, Krugman P, Venables A (1999) The Spatial Economy: Cities, Regions and International Trade. Cambridge, MA: MIT Press.

[28] Henderson JV (1977) Economic Theory and the Cities. New York: Academic Press.

[29] Bettencourt LMA, Samaniego H, Youn H (2013) Professional Diversity and the productivity of cities. Available: http://arxiv.org/abs/1210.7335. 10.1038/srep05393 PMC4066264 24953448

[30] Smith ME (1992) Archaeological Research at Aztec-period Rural Sites in Morelos, Mexico. Pittsburgh: University of Pittsburgh, Department of Anthropology.

[31] Millon R, Drewitt RB, Cowgill GL (1973) The Teotihuacan Map. Part Two: Maps. Austin: University of Texas Press.

[32] Nowak M (2006) Five Rules for the evolution of cooperation. Science 314: 1560–1563. 17158317 10.1126/science.1133755 PMC3279745

[33] Marshall A (1890) Principles of Economics. London: MacMillan.

[34] World Basemap (ESRI, Delorme, NAVTEQ, 2012).

[35] USGS, NASA/JAXA, GLCF. ASTER Imagery (USGS, GLCF, Sioux Falls, 2005).

[36] Kempes CP, West GB, Crowell K, Girvan M (2011) Predicting Maximum Tree Heights and Other Traits from Allometric Scaling and Resource Limitations. PLoS ONE 6 (6) e20551 doi:10.1371/journal.pone.0020551 21695189 10.1371/journal.pone.0020551 PMC3113805

[37] Parsons JR (1971) Prehistoric Settlement Patterns in the Texcoco Region, Mexico. Ann Arbor: Museum of Anthroplogy, University of Michigan.

[38] Parsons JR, Brumfiel E, Parsons M, Wilson D (1982) Prehispanic Settlement Patterns in the Southern Valley of Mexico: The Chalco-Xochimilco Region. Ann Arbor: Museum of Anthropology, University of Michigan.

[39] Parsons JR, Kintigh KW, Gregg S (1983) Archaeological Settlement Pattern Data from the Chalco, Xochimilco, Ixtapalapa, Texcoco and Zumpango Regions, Mexico. Ann Arbor: Museum of Anthropology, University of Michigan.

[40] Parsons JR (2008) Prehispanic Settlement Patterns in the Northwestern Valley of Mexico: The Zumpango Region. Ann Arbor: Museum of Anthropology, University of Michigan.

[41] Blanton RE (1972) Prehispanic Settlement Patterns of the Ixtapalapa Penninsula Region, Mexico. University Park: The Pennsylvania State University.

[42] Sanders WT, Gorenflo LJ (2007) Prehispanic Settlement Patterns in the Cuautitlan Region, Mexico. University Park: The Pennsylvania State University.

[43] Gorenflo LJ, Sanders WT (2007) Archaeological Settlement Pattern Data from the Cuautitlan, Temascalapa, and Teotihuacan Regions, Mexico. University Park: The Pennsylvania State University.

[44] Sanders WT (1987) The Teotihuacan Valley Project Final Report - Volume 4: The Toltec Period Occupation of the Valley, Part 2: Surface Survey and Special Studies. University Park: The Pennsylvania State University.

[45] Sanders WT (1996) The Teotihuacan Valley Project Final Report - Volume 3: The Teotihuacan Period Occupation of the Valley, Part 3: The Surface Survey. University Park: The Pennsylvania State University.

[46] Evans ST, Sanders WT (2000) The Teotihuacan Valley Project Final Report Volume 5: The Aztec Period Occupation of the Valley, Part 1 - Natural Environment, 20th Century Occupation, Survey Methodology, and Site Descriptions. University Park: The Pennsylvania State University.

[47] Sanders WT (1975) The Teotihuacan Valley Project Final Report - Volume 2: The Formative Period Occupation of the Valley, Part 1: Texts and Tables. University Park: The Pennsylvania State University.

[48] Millon R (1973) The Teotihuacan Map. Part One: Text. Austin: University of Texas Press.

[49] Smith ME (2008) Aztec City-State Capitals. Gainesville: University Press of Florida.

[50] Cowgill GL (1997) State and Society at Teotihuacan, Mexico. Annual Review of Anthropology 26: 129–161.

[51] Clauset A, Shalizi CR, Newman MEJ (2009) Power-law distributions in empirical data. SIAM Review 51 (4) 661–703.

[52] Chernick MR (1999) Bootstrap Methods: A Practitioner's Guide. New York: John Wiley & Sons, Inc.

[53] Sanders WT (1976) In The Valley of Mexico: Studies in Pre-Hispanic Ecology and Society. Wolf ER, editor. Albuquerque: University of New Mexico Press. pp. 101–159.

[54] Sanders WT (1965) The Cultural Ecology of the Teotihuacan Valley: A Preliminary Report of the Results of the Teotihuacan Valley Project. Department of Sociology and Anthropology, The Pennsylvania State University.

[55] Smith ME (2005) City Size in Late Postclassic Mesoamerica. Journal of Urban History 31 (4) 403–434.

[56] Trigger BG (2003) Understanding Early Civilizations. Cambridge: Cambridge University Press.

[57] Smith ME, Feinman GM, Drennan RD, Earle T, Morris I (2012) Archaeology as a Social Science. Proceedings of the National Academy of Science of the USA 109 (20) 7617–7621. 10.1073/pnas.1201714109 PMC3356624 22547811

[58] Wright HT (2001) Cultural Action in the Uruk World. Uruk Mesopotamia and its Neighbors. Rothman MS, editor. Santa Fe: School of American Research Press. pp 123–148.

[59] Drennan RD (1984) Long-Distance Transport Costs in Pre-Hispanic Mesoamerica. American Anthropologist 86 (1) 105–112.

[60] Arnauld MC, Manzanilla LR, Smith ME, editors (2012) The Neighborhood as a Social and Spatial Unit in Mesoamerican Cities. Tucson: University of Arizona Press.

[61] Bettencourt LMA, Lobo J, Strumsky D, West GB (2010) Urban Scaling and Its Deviations: Revealing the Structure of Wealth, Innovation and Crime across Cities. PLoS ONE 5 (11) e13541. 21085659 10.1371/journal.pone.0013541 PMC2978092

